# Genetic Variation in a Crossing Population of *Camellia oleifera* Based on ddRAD Sequencing and Analysis of Association with Fruit Traits

**DOI:** 10.3390/cimb47020092

**Published:** 2025-01-31

**Authors:** Lexin Zhou, Yu Li, Ling Ye, Jiani Li, Tian Liang, Yanxuan Liu, Weiwei Xie, Yiqing Xie, Shipin Chen, Hui Chen

**Affiliations:** 1 College of Forestry, Fujian Agriculture and Forestry University, Fuzhou 350002, China; zlx2762190752@163.com (L.Z.); yeling@fafu.edu.cn (L.Y.); lijiani2016@163.com (J.L.); liangt_forest@163.com (T.L.); lyxsjdy@163.com (Y.L.); weiweixie2020@163.com (W.X.); fjcsp@126.com (S.C.); huichenfafu@163.com (H.C.); 2 Fujian Academy of Forestry, Fuzhou 350012, China; fjly168@sina.com; 3 The Oil Tea Engineering Technological Research Center of Fujian Province, Fuzhou 350002, China

**Keywords:** *Camellia oleifera*, crossing population, genetic variation, association analysis, SNP

## Abstract

Tea oil is an important high-quality edible oil derived from woody plants. *Camellia oleifera* is the largest and most widely planted oil-producing plant in the *Camellia* genus in China, and its seeds are the most important source for obtaining tea oil. In current research, improving the yield and quality of tea oil is the main goal of oil tea genetic breeding. The aim of this study was to investigate the degree of genetic variation in an early crossing population of *C. oleifera* and identify single nucleotide polymorphisms (SNPs) and genes significantly associated with fruit traits, which can provide a basis for marker-assisted selection and gene editing for achieving trait improvement in the future. In this study, we selected a crossing population of approximately 40-year-old *C. oleifera* with a total of 330 samples. Then, ddRAD sequencing was used for SNP calling and population genetic analysis, and association analysis was performed on fruit traits measured repeatedly for two consecutive years. The research results indicate that over 8 million high-quality SNPs have been identified, but the vast majority of SNPs occur in intergenic regions. The nucleotide polymorphism of this population is at a low level, and Tajima’s D values are mostly greater than 0, indicating that the change in this population was not suitable for the model of central evolution. The population structure analysis shows that the population has seven theoretical sources of genetic material and can be divided into seven groups, and the clustering analysis results support the population structure analysis results. Association analysis identified significant SNPs associated with genes related to the seed number of a single fruit and seed kernel oil content. Our findings provide a basis for molecular breeding and future genetic improvement of cultivated oil tea.

## 1. Introduction

*Camellia* is a large genus in the family Theaceae of the dicotyledonous plant order in the plant kingdom. There are 280 species in the entire genus, about 240 of which are distributed in China [1,2]. The plants in this genus have high economic benefits and value. A species with tender leaves that can be brewed for drinking is the popular *C. sinensis*. Species that can be extracted for tea oil with a high seed oil content are commonly referred to as “oil tea” plants, such as *C. oleifera*, *C. drupifera*, *C. meiocarpa*, *C. chekiangoleosa*, *and C. semiserrata*. In China, *C. oleifera* is the most common and widely cultivated species. Its cultivation area is distributed in the Yangtze River basin, the Pearl River basin, and other southern regions of China [3]. The history of recording tea and fruit oil harvesting in China can be traced back to the late Yuan and early Ming dynasties [3], and artificial domestication began in the late Ming Dynasty [4]. Tea oil is rich in unsaturated fatty acids and non-saponifiable substances, which give it excellent anti-inflammatory, antibacterial, and other functions, which play an important role in human health [5,6,7,8,9,10].

The seeds of *C. oleifera* have a high oil content and are the main source of tea oil. Therefore, improving the yield and quality of tea oil is the main goal of genetic breeding research in *C. oleifera*. Since the 1970s, there has been extensive interest in the artificial cultivation and breeding of *C. oleifera* in China. After nearly 50 years of breeding work, there are now over 360 varieties or clones of this species. Early breeding was mostly carried out by directly selecting excellent phenotypic traits, and efforts were made towards achieving high yield, large fruit, high oil content, and high unsaturated fatty acids. Later, cross-breeding gradually became an important means of breeding work. After long-term breeding development, the oil content of *C. oleifera* seeds can reach over 58% [11], and the oleic acid content in tea oil can reach over 76% [12]. The oleic acid, linoleic acid, and other indicators of *C. oleifera* cultivated populations (asexual populations) are also significantly higher than those of non-cultivated populations [13]. The breeding and promotion of excellent varieties of *C. oleifera* have greatly improved the yield and quality of tea oil.

However, genetic approaches to guiding *C. oleifera* breeding have been hampered by the complexity of its chromosomal structure. The chromosome base of *Camellia* species is constant (*n* = 15), but the chromosome ploidy of species within the genus is not uniform [14], and even the ploidy of different varieties within the species varies. There are diploid, tetraploid, and hexaploid species in the genus *Camellia* [3,15], and the commonly cultivated *C. oleifera* is mainly hexaploid. Tetraploid *C. oleifera* is sometimes classified as a variant (*C. oleifera* var. *monosperma*) [16] due to its similar genetic background but unique phenotypic characteristics, and others consider it a separate species of *C. meiocarpa*. The complex chromosomal ploidy and genetic background may be a major obstacle to the research progress of *C. oleifera*. The first *C. oleifera* genome, which was released in 2022 [17], was derived from a variant (*C. Oleifera* var. “*Nanyongensis*”, CON) diploid (2*n* = 2*x* = 30) plant speculated to be the wild ancestor of *C. oleifera*. The publication of this genome provides favorable conditions for the better development of *C. oleifera* gene resources.

The mechanism of genes determining important traits is helpful for breeding, and many studies have focused on gene control of fat synthesis [18,19,20]. However, yield and quality are complex quantitative traits, and limited genomic resources and complexities of polyploidy make the breeding of oil tea extremely challenging. The plant material selected for this study was a crossing population that formed relatively early and was established in the early stages of *C. oleifera* breeding, having the genetic background of early *C. oleifera* populations to some extent. In selecting this population, the aims were to elucidate the degree of genetic variation in the early breeding population and identify related genes that are significantly associated with fruit traits, thus providing a basis for molecular breeding and future genetic improvement of *C. oleifera*.

## 2. Materials and Methods

### 2.1. Plant Materials

The material used in this study was a crossing population obtained with complete double-row crossing (excluding self-crossing) of 10 parents, with a total of 25 combinations and 330 samples. The group adopted a balanced incomplete block design (BIBD) for planting, with a total of 48 treatments and one contrast. The 48 treatments were obtained using grading seedlings from 25 crossing combinations, and the blank contrast was the half-sib offspring of the parents. The block size was seven, the number of repetitions was eight, and the number of encounters was one (Table S1). Ten parents were selected as excellent clones in 1975, and the crossing was carried out in 1981. The seeds obtained in 1982 were first sown and then propagated for afforestation in 1984. The age of the crossing population is 40 years.

### 2.2. DNA Extraction

The genomic DNA of each individual was extracted using “Broad spectrum plant genomic DNA rapid extraction kit” (Beijing Bomeid Gene Technology Co., Ltd., Beijing, China, http://www.biomed168.com, accessed on 25 February 2023) and detected with a microspectrophotometer (Nano-200). The absorbance (A260/A280) of the DNA was between 1.8 and 2.0, indicating that the quality met the standard. Only DNA that met quality standards was used for subsequent sequencing.

### 2.3. ddRAD-Seq

Restriction endonucleases Sac 1 and Mse 1 were used to cleave the genomic DNA of each sample. The target genome was cut into long and short DNA fragments with sticky ends, and the digested products were purified. Then, R1 and R2 adapters were attached to both ends of the sequence fragment, and the spliced product was purified. Then, we selected 150 bp fragments for PCR amplification and introduced an individual identification tag index near the enzyme cleavage site at the second end to enable more samples to be mixed for sequencing. Finally, we used an Illumina platform for sequencing to obtain raw data.

The filtering method included using the fastqc (version 0.11.5) and fastp (version 0.20.0) software [21], setting parameters to filter the data to remove low-quality sequencing data, and finally obtaining clean data. The above sequencing and quality control were completed by Wuhan Igenebook Biotechnology Co., Ltd. (Wuhan, China, http://www.igenebook.com, accessed on 25 February 2023).

### 2.4. SNP Calling

In the Linux environment, BWA (v07.17) [22] and GATK software (v4.2.4.1) [23] were used to perform SNP detection and genotyping based on the CON genome as a reference. We filtered the mutation sites in the GATK environment, using the GATK software’s “QD < 2.0, FS > 60.0, QUAL < 30.0, MQ < 40.0, MQRankSum < −12.5, Read-PosRankSum < −8.0, SOR > 3.0” as the filtering criteria to remove low-quality SNP sites, and only retained double allele sites. In the snpEff software (v4.3) [24], the gff annotation file in the genome was used to annotate SNPs, determine the mutation regions, and count the number of various SNPs.

### 2.5. Genetic Diversity Analysis

Using Plink software (v1.90b6.21) [25], the SNPs filtered using GATK were filtered once again using the standard of retaining sites with deletion rates less than 0.1 and allele frequencies greater than 0.01. Then, based on VCFtools software (v0.1.17) [26], the nucleotide diversity index (π) and Tajima’s D of the 330 genotypes were calculated, and a Manhattan plot was plotted in R (v4.3.0).

### 2.6. Population Structure Analysis

Firstly, the SNPs filtered using Plink were subjected to linkage disequilibrium (LD) filtering again. Then, ADMIXTURE software (v1.3.0) was used to estimate the number of ancestors based on the model [27]. The range of k values was set to 2–10, the CV error values for each K value were calculated separately, and a structure graph was plotted using R. The Tassel software (v5.2.40) was used to format the file storing SNP information [28]; then, FastTree software (v2.1.11) was used to construct a tree using the approximate maximum likelihood method [29], and the iTOL website (https://itol.embl.de, accessed on accessed on 25 February 2023) was used to implement tree visualization [30].

### 2.7. Association Analysis of Fruit Traits

The fruit traits of 330 genotypes were measured, including fruit height, fruit width, fruit weight, pericarp thickness, seed number of a single fruit, fresh seed weight of a single fruit, dry seed weight of a single fruit, dry kernel weight of a single fruit, and oil content of dried kernels. Phenotypic data were measured in 2020 and 2021 (Table S2), and data from each year were analyzed independently.

Using association analysis methods, we searched for SNPs closely related to fruit traits. Firstly, Beagle software (v5.4) was used to infer the genotype of population SNPs [31], to compensate for the lack of shallow sequencing depth. Then, EMMAX software (beta-7 March 2010) was used to calculate the correlation between each SNP and fruit trait based on an approximate mixed linear model (mlm) [32], and the result was visualized in R. Based on SNPs significantly associated with fruit traits, we located relevant genes and plotted them in LDBlockShow software (v1.39) [33].

## 3. Results

### 3.1. SNP Calling and Functional Annotation

Sequencing the 330 genotypes generated a total of 2,274,061,170 clean reads, with an average of 6.89 million pairs of reads per genotype. The sites with sequencing quality meeting Q20 and Q30 standards account for 97.36% and 92.88% of the total sites, respectively. And the average GC content was approximately 42.44% (Table S3). The number of SNPs filtered with GATK was 11,035,516. The number of SNPs filtered by deletion rate and allele frequency was 8,324,848. After LD filtering, the number of SNPs was 282,810.

The results of SNP annotation (Table 1) indicated that about 88% of SNPs were located in intergenic regions, with missense mutations, loss of start codons, and early acquisition of stop codons as equivariant sites. These sites, which have a significant impact on transcription, translation, and other processes in plant cells, accounted for a relatively small proportion of the overall population, about 2% of all sites.

### 3.2. Genetic Diversity

**Figure 1:**
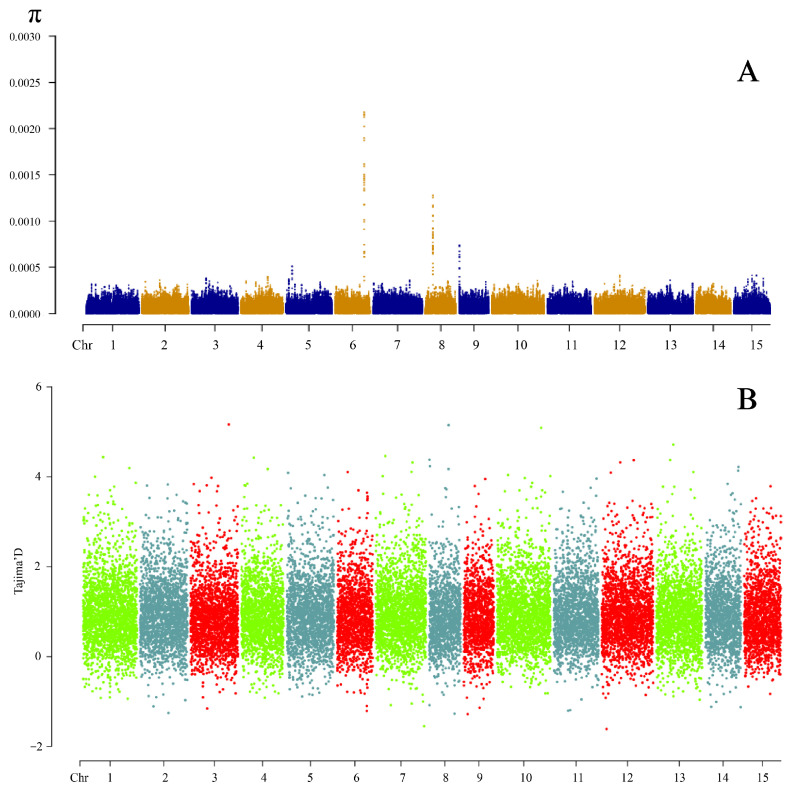
Genetic diversity of the *C. oleifera* crossing population: (**A**) nucleotide diversity index and (**B**) Tajima’s D value.

In order to understand the degree of genetic variation in this population, we analyzed the nucleotide polymorphism and D value of the population. The values of the nucleotide diversity index (π) for the 330 genotypes are shown in Figure 1A, which indicates that the nucleotide diversity is at a low level. Most of the polymorphic loci exhibited π values of less than 0.0005, and only some regions on chromosomes 6, 8, and 9 exhibit high diversity. From Figure 1B, it can be seen that the Tajima’s D values of the population are mostly greater than 0, which indicates that the group is not suitable for the theory of central evolution.

### 3.3. Population Structure

In order to explore the population structure of 330 genotypes in the population, we used ADMIXURE to calculate the optimal K value of the population and drew a structure matrix graph. At the same time, we used Fasttree for cluster analysis to synchronously support the results of the previous analysis.

**Figure 2:**
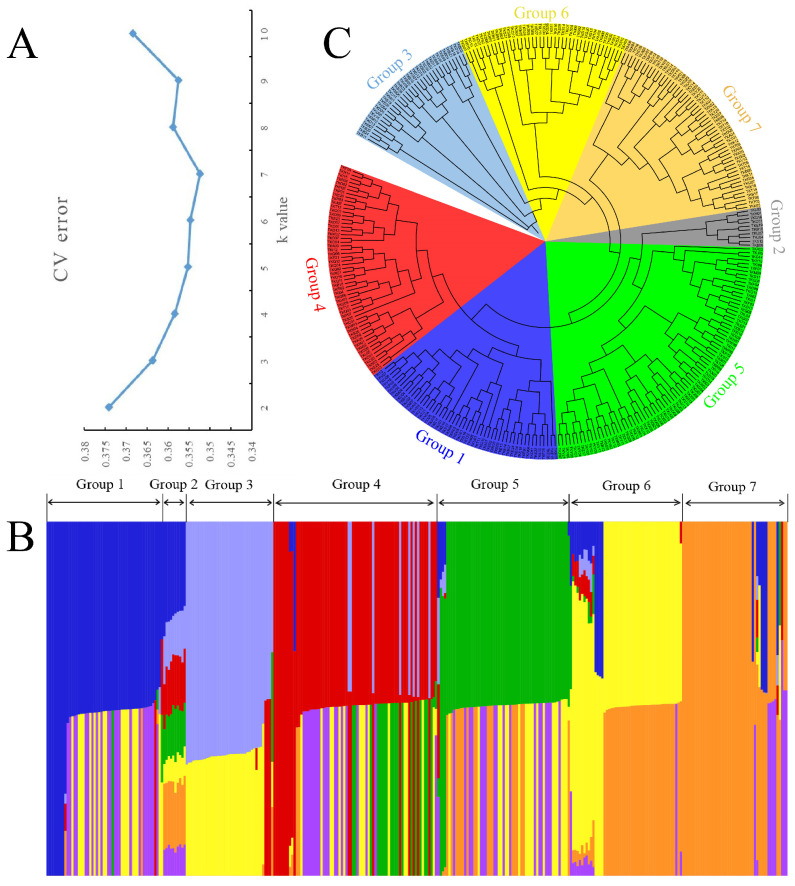
Population structure of the *C. oleifera* crossing population. (**A**) CV error values under different K value conditions. (**B**) Genetic structure matrix of the *C. oleifera* crossing population (when K = 7). (**C**) Cluster analysis of 330 *C. oleifera* genotypes based on approximate maximum likelihood method.

When calculating the value of K, we set the value of K to 2–10 and calculated each value separately. The results showed that the CV error was the lowest when K = 7 (Figure 2A), indicating that the best theoretical genetic material source for this population is 7.

Subsequently, we plotted a matrix graph (Figure 2B) based on the sequence matrix obtained by calculating K = 7 and found that 330 *C. oleifera* genotypes were divided into seven groups. Group 1 mainly included the families TKE and TKV. Group 2 was most unique, as it had seven sources of genetic material, based on the structure diagram. Group 3 mainly included the families TKB, TKD, and TKM. Group 4 included the families TKC, TKN, TKO, TKP, and TKQ, all of which share the common trait of having M05 parents. Group 5 included the families TKG, TKR, TKU, TKX, and TKY, all of which had M10 parents in common. Group 6 was mainly composed of individuals from the TKS family. Group 7 mainly included individuals from the TKK and TKT families, which were obtained by crossing the parents M03 and M08 in a reciprocal cross. The results of cluster analysis support population structure analysis because their classification results are the same (Figure 2C). From the classification results, it can be seen that the classification of 330 genotypes is not strictly distinguished according to the differences in families. Individuals in the branches have similar characteristics, such as at least one identical maternal or paternal parent in their parents.

### 3.4. Association of Fruit Traits with SNPs

In order to search for loci and genes related to the development of *C. oleifera* fruit traits, we used an approximate mixed linear model based on EMMAX to calculate the correlation between fruit traits and SNPs of 330 genotypes. A total of 28 SNPs were found to be significantly associated with fruit traits (Table S4), and each SNP corresponds to only one trait, but there are multiple SNPs at different positions corresponding to the same trait.

**Figure 3:**
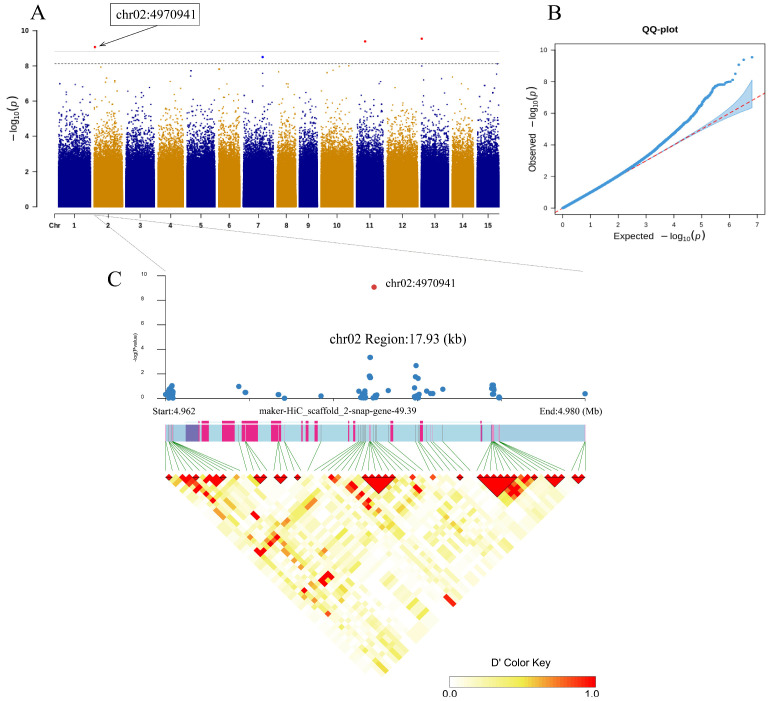
Association of ‘seed number of a single fruit’ (2021) with SNPs. (**A**) Manhattan plot showing the degree of association between SNPs and ‘seed number of a single fruit’ on 15 chromosomes. We evaluated the correlation at scales of 0.01 and 0.05, where the threshold line is equal to 0.01/0.05 divided by the number of SNPs, and then took the negative logarithm. The dashed and solid lines represent the threshold lines at scales of 0.05 and 0.01, respectively. (**B**) Correction QQ-plot for association analysis of ‘seed number of a single fruit’. (**C**) LD block (17.93 kb) in the region of gene “maker-HiC_scaffolds 2-snap-gene-49.39”.

**Figure 4:**
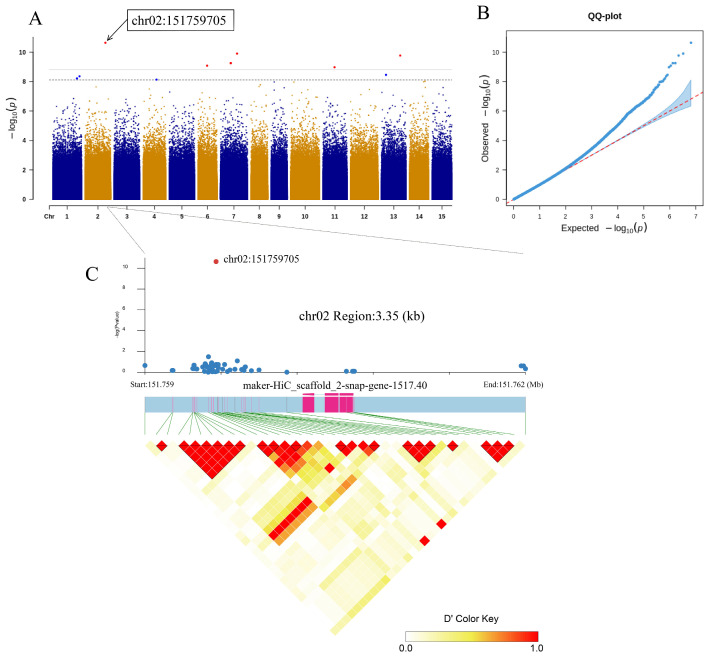
Association of ‘oil content of dried kernels’ (2021) with SNPs. (**A**) Manhattan plot showing the degree of association between SNPs and ‘oil content of dried kernels’ on 15 chromosomes. We evaluated the correlation at scales of 0.01 and 0.05, where the threshold line is equal to 0.01/0.05 divided by the number of SNPs, and then took the negative logarithm. The dashed and solid lines represent the threshold lines at scales of 0.05 and 0.01, respectively. (**B**) Correction QQ-plot for association analysis of ‘oil content of dried kernels’. (**C**) LD block (3.35 Kb) in the region of gene “maker-HiC_scaffolds 2-snap-gene-1517.40”.

Among the 28 SNPs, 26 were located in the intergenic region, 1 was located in the intron region, and 1 SNP was located upstream of the gene. The SNP located in the intron region (chr02:4970941) was significantly associated with the seed number of a single fruit (2021) (Figure 3A,B), and the SNP located in the intergenic region (chr02:151759705) was significantly associated with seed kernel oil content (2021) (Figure 4A,B).

In previous studies, it was found that the LD decay distance of *C. oleifera* populations is usually between 1 and 2 kb [17,34], so we referred to these research results to plot the areas where chr02:4970941 and chr02:151759705 are located. The size of the plot interval where chr02:4970941 is located was 17.93 kb (Figure 3C), and the ID of the significantly associated gene was “marker HiC_scaffolds 2-snap-gene-49.39”. It could be clearly seen from the graph that the length of this gene was longer, but the exon region was shorter and more dispersed. After comparing with the annotation file, we found that the gene has two transcription modes, with the same promoter and terminator, but different base positions in the 3′UTR and 5′UTR in each transcript. The size of the plot interval where chr02:151759705 is located was 3.35 kb (Figure 4C), and the ID of the significantly associated gene was “marker HiC_scaffolds 2-snap-gene-1517.40”. This gene has a shorter length and only one transcription mode. In Figure 3C and Figure 4C, we found that the degree of linkage between SNPs and genes is not tight, which may be due to shallow sequencing depth resulting in less site coverage.

In order to further validate the function of the genes associated with the two traits, we used TBtools (v2.07) [35] software to extract the protein sequences of the two genes and performed a search and alignment in NCBI (National Center for Biotechnology Information). The two protein sequences of “marker HiC_scaffolds 2-snap-gene-49.39” showed little difference, and the comparison results showed that the protein encoded by this gene had high homology with Calmodulin-binding transcription activator 5 (*CAMTA*5) and some hypothetical protein sequences in *C. lanceoleosa* (Accession: KAI8010449.1) [36] and *C. sinensis* (Accession: XP_028094567.1). The protein sequence alignment results of “marker HiC_scaffolds 2-snap-gene-1517.40” are relatively few, and they correspond to a certain hypothetical protein in *C. sinensis*. The similarity between the two is also not high. The structure of the protein encoded by this gene has not been determined yet, so we cannot infer the function that this gene performs.

## 4. Discussion

We studied the genetic variation of an early artificial population of *C. oleifera* and conducted an association analysis between fruit traits and SNPs. The genetic diversity was relatively low, and the population structure was basically consistent with the distribution of individuals in the crossing combinations. An association analysis identified two candidate genes related to the number of seeds and oil content of dried kernels, which need further in-depth identification.

In this research, the total number of SNPs in the population was large, but after SNP annotation, the variation of most loci occurred in less affected areas. The nucleotide polymorphism also showed that the genetic diversity index of this population was very low, significantly lower than other populations of the related species or closely related populations of the same genus [34,37]. The D values showed that most of them were greater than 0, indicating that the number of polymorphic loci in this population was very small [38]. The population was not in accordance with neutral evolution theory, and this result was consistent with the source of the population. The genetic composition of this *C. oleifera* population only came from the parents, who represent a small component of its initial population. The artificial population in this study developed from a small portion of the original population and has low genetic diversity, which is consistent with the genetic dynamics of the population after experiencing bottlenecks [39]. So, the breeding population has lower genetic variation and experienced strong artificial selection, and future breeding should focus on maintaining suitable genetic diversity.

The research population in this study was relatively unique because there was a hybrid design in the population, and each genotype had known parents. According to the calculation of the optimal K value for the population, the theoretical number of genetic material sources for this population was 7, which was inconsistent with the known number of 10 parents. This result may indicate that some of the genetic material for these 10 parents comes from the same ancestor. The analysis results of population structure analysis and cluster analysis both supported the division of this group into seven groups, and the classification results are not strictly based on family lineage, but on whether individuals have the same father or mother. In the classification of subgroups, we found that a few individuals are distributed in branches that theoretically do not belong to them, for example, Group 7 was the main subgroup of TKK and TKT families, while TKT13 was distributed in Group 5 and TKK08 was distributed in Group 1. The reason for this may be due to contamination of pollen sampling during artificial hybridization, leading to pollination errors. In the population genetic structure map, we can also find that the proportion of genetic material composition in most individuals follows a 1:1 rule. From the perspective of artificial hybridization, this phenomenon is easy to explain because the genetic composition of offspring comes from both parents and is difficult to be influenced by other factors. This result may occur when there are significant genetic differences between the father and mother.

The LD decay distance of a group is influenced by the nature of the group itself. The LD decay distance of domesticated groups is longer than that of non-domesticated groups [37]. Groups with fast generational changes often have shorter LD decay distances than those with slow generational changes, as demonstrated by studies on silkworms and wild silkworms [40]. Because the material used in this study is hexaploid but the reference genome is diploid, the situation of SNP cannot truly reflect the true distribution on chromosomes and, therefore, cannot reflect the true LD attenuation situation of the population. Thus, the selection of LD decay distance in this study referred to the results of other researchers.

In the association analysis, we preliminarily identified two SNPs that were related to the seed number of a single fruit and the seed kernel oil content, and within the range of LD decay, these two SNPs are associated with genes. Due to the fact that the seed kernel was the most important part for oil accumulation in *C. oleifera* fruits, for the trait of “seed number of a single fruit”, we hope that the fewer seeds per fruit, the better. This is because in a limited growth space, the fewer seeds there are, the more space for the seed kernel to grow, which was most beneficial for the production of tea oil. In the comparison results, “maker-HiC_scaffolds 2-snap-gene-49.39” has a high degree of homology with the gene encoding *CAMTA*. *CAMTA* is believed to exist in most plant species, such as sugarcane [41], sesame [42], wheat [43], tea [44], etc. When plants respond to biotic or abiotic stress, this factor plays an important role [45], and it is also associated with plant growth and development [46]. In their research on Arabidopsis, Li et al. [47] showed that both *AtCAMTA1* and *AtCAMTA5* are expressed during pollen development, which can affect fruit growth and development. *Yang* et al. [48] found that in tomatoes, the *CAMTA*/*SR* homologous gene *SISR* regulates the mediation of ethylene and calcium, thereby regulating the fruit development process. It can be inferred that CAMTA plays a corresponding role in plant stress response and fruit development. However, there were currently no reports on the related genes controlling seed quantity in *C. oleifera* research. In this study, there was a high correlation between the gene “maker-HiC_scaffolds 2-snap-gene-49.39” and the trait of seed quantity, and after comparison, this gene showed high homology with the gene encoding *CAMTA*. Therefore, it can be preliminarily inferred that this gene may affect the development of *C. oleifera* fruit by encoding related proteins, thereby regulating the number of seeds in fruit. If this gene can regulate the number of seeds, it will play an important role in *C. oleifera* breeding. However, how this gene controls the seed number is still unknown, and we will continue to study the gene functions in future. For the trait of “oil content of dried kernels”, a higher oil content means more oil accumulation. In previous studies, there have been some research results on the mechanism of tea oil accumulation, which was a relatively complex metabolic process involving multiple genes [49]. Among them, genes such as SADS, FAD2, GPD1, and DGAT play important roles in the metabolism of oil [18,19,20]. In subsequent research, further exploration of the functions of the two genes obtained in this study should be conducted, and their respective mechanisms of action should be elucidated.

## Figures and Tables

**Table 1:** Annotation information for SNPs.

Variant Type	SNP Number	Ratio
3′ UTR variant	15,026	0.1680%
5′ UTR premature start codon gain variant	1920	0.0210%
5′ UTR variant	11,879	0.1330%
Downstream gene variant	258,378	2.8870%
Initiator codon variant	24	0.0000%
Intergenic region	7,875,582	87.9850%
Intron variant	307,504	3.4350%
Missense variant	124,931	1.3960%
Splice acceptor variant	722	0.0080%
Splice donor variant	541	0.0060%
Splice region variant	8671	0.0970%
Start lost	253	0.0030%
Start retained variant	2	0.0000%
Stop gained	5506	0.0620%
Stop lost	308	0.0030%
Stop retained variant	132	0.0010%
Synonymous variant	85,293	0.9530%
Upstream gene variant	254,377	2.8420%
Total	8,951,049	100%

## Data Availability

The ddRAD-seq data are available in the Genome Sequence Archive (GSA) of the NGDC (National Genomics Data Center) under accession number PRJCA030902 (CRA019635). The other original contributions presented in this study are included in the article/Supplementary Materials, and further inquiries can be directed to the corresponding author (Yu Li).

## Supplementary Materials

The following supporting information can be downloaded at: www.mdpi.com/article/10.3390/cimb47020092/s1. Table S1. Crossing combination and block design information; Table S2. Fruit trait information; Table S3. Sequencing quality information; Table S4. The SNP information significantly associated with fruit traits.
